# Closely related ants exhibit species-specific transcriptional responses during temperature acclimation

**DOI:** 10.1093/g3journal/jkag078

**Published:** 2026-05-06

**Authors:** Barbara Feldmeyer, Florian Menzel

**Affiliations:** Molecular Ecology, Senckenberg Biodiversity and Climate Research Centre, Georg-Voigt-Straße 14-16, Frankfurt am Main 60325, Germany; Institute of Organismic and Molecular Evolution (iomE), Johannes Gutenberg-University Mainz, Hanns-Dieter-Hüsch-Weg 15, Mainz 55128, Germany

**Keywords:** Formicidae, acclimation, temperature response, cuticular hydrocarbons, climate change, gene expression, RNA-seq

## Abstract

Ambient temperature is a major selective pressure, affecting traits from development to survival. In insects, cuticular hydrocarbons (CHCs) reduce water loss and can be adjusted during acclimation; however, the molecular basis and evolutionary conservation of this plasticity remain poorly understood. We examined transcriptional responses of 3 congeneric *Lasius* ant species from distinct habitats acclimated to 2 constant and 1 fluctuating temperature regimes. We analyzed global gene (co-)expression patterns and candidate genes involved in CHC biosynthesis. All acclimation treatments induced changes in gene expression, with fluctuating temperatures eliciting the fewest. The arboreal *Lasius brunneus* showed the weakest response and uniquely lacked increased desiccation resistance, indicating lower plasticity relative to *Lasius niger* and *Lasius platythorax*. Coexpression networks revealed that CHC-related genes were broadly distributed across modules, whereas global expression patterns were highly conserved across species. These results highlight species-specific plasticity within a conserved transcriptional framework, with implications for resilience to climate change.

## Introduction

Faced with climate change, organisms must respond to increasingly variable conditions through processes operating on different timescales, including genetic adaptation and phenotypic plasticity. While genetic adaptation involves heritable changes across generations, phenotypic plasticity, such as acclimation, allows individuals to adjust trait expression within their lifetime (Pigliucci 2001; Angilletta 2009; Chown et al. 2011).

Acclimation allows coping with variation in temperature and humidity, enhancing drought resistance, heat tolerance, and survival (Hoffmann and Watson 1993; Chown et al. 2011; Baumgart et al. 2022). The underlying physiological mechanisms include altered metabolism, stress protein expression, and osmolyte synthesis (Chown and Terblanche 2006; van Dooremalen et al. 2011). Responses often differ between constant and fluctuating regimes, with fluctuating conditions frequently enhancing tolerance and performance (Peng et al. 2014; Salachan and Sørensen 2022). Thus, plasticity may buffer organisms against climate change (Seebacher et al. 2015).

Cuticular hydrocarbons (CHCs) cover the cuticle of all insects, serving as a desiccation barrier and a communication signal (Blomquist and Bagnères 2010). Acclimatory CHC changes maintain CHC viscosity and reduce water permeability (Huthmacher et al. 2025). CHC acclimation to warm conditions is associated with increased heat or drought tolerance (Menzel et al. 2018; Baumgart et al. 2022). CHC profiles are highly complex and species-specific, often comprising more than 100 compounds (Sprenger and Menzel 2020). Consequently, acclimatory CHC changes differ among species, although overall patterns are similar. Warm conditions generally lead to upregulation of *n-*alkanes and downregulation of dimethyl and trimethyl alkanes in *Lasius* and other ant species (Sprenger et al. 2018; Baumgart et al. 2022). However, whether the molecular mechanisms underlying acclimation are conserved or evolved independently remains largely unknown.

Here, we investigated acclimatory gene expression in 3 congeneric ant species with similar life histories but contrasting microhabitats: *Lasius niger*, inhabiting sun-exposed, open meadows; its sister species *Lasius platythorax* (Seifert 1991), living on the ground of forests; and the phylogenetically more distant, arboreal *Lasius brunneus* (Maruyama et al. 2008; Seifert 2018). A previous study showed species-specific, but similar CHC changes following acclimation to constant and fluctuating temperatures, with enhanced desiccation resistance in *L. niger* and *L. platythorax*, but not in *L. brunneus* (Baumgart et al. 2022).

Using transcriptomic data, we investigated the molecular basis of temperature acclimation. We tested how acclimatory responses differ among species in number, identity, function, and coexpression of differentially expressed genes. First, we predicted the highest transcriptional plasticity in *L. niger*, which occupies open habitats with large daily temperature variation (Renaud et al. 2011), and the lowest in *L. brunneus*, where acclimation had limited phenotypic effects (Baumgart et al. 2022). Across species, we secondly predicted that fluctuating temperature regimes induce stronger transcriptional responses than constant regimes, consistent with the increased regulatory demands imposed by thermal variability (Podrabsky and Somero 2004). Third, we analyzed the expression of CHC biosynthesis genes, predicting species-specific expression changes, but coexpression of CHC-candidate genes associated with temperature regime, given their role in thermal acclimation. Fourth, we compared transcriptome-wide acclimation responses between species and predicted partial conservation of gene coexpression networks, reflecting shared regulatory architectures shaped by common ancestry.

## Material and methods

### Study species

Ants from 12 colonies each of *L. niger, L. platythorax, and L. brunneus* (Hymenoptera: Formicidae) were collected in May 2019 in the vicinity of Mainz, Germany (*L. niger*: meadows around the University of Mainz; *L. platythorax*: forest floor of the Ober-Olmer Wald; and *L. brunneus*: at tree trunks in the Gonsenheimer Wald). All 3 species occur throughout Europe with largely similar distribution ranges from Spain in the Southwest to mid-Scandinavia as northernmost and Turkey as easternmost confirmed site (AntWiki 2026). Thus, all 3 species have populations with annual mean temperatures from 2 °C (mid-Scandinavia) to 14 °C (Spain) and daily temperature fluctuations from 4 °C (Denmark, England) to 12 °C (Spain, Romania) (Fick and Hijmans 2017).

We collected entire nests for *L. niger* and *L. platythorax*, but only worker groups of *L. brunneus* due to their hard-to-access arboreal nests. From each of these colonies, we created 3 worker groups consisting of 20 foragers, 20 nurses, and at least 5 brood items where possible, and no queens. Each of the 3 worker groups per colony was designated for 1 of the 3 climate treatments. See Baumgart et al. 2022 for more details.

### Acclimation treatments and RNA sequencing

Ant worker groups were kept under 3 different temperature treatments: 20 °C, 28 °C, and a fluctuating regime, each with a 12h:12 h light/dark cycle. The fluctuating regime had 20 °C at night (8 h) and 28 °C during the day (8 h), with 4 h ramps in between. The treatments were maintained in climate cabinets (Rubarth Apparate GmbH, Frankfurt am Main, Germany). The individual boxes had a plaster floor that was regularly watered to ensure a humidity of nearly 100%. The boxes were covered with lids and sealed with parafilm to ensure constant humidity. Honey, dead crickets, and water in Eppendorf cups with a cotton plug were provided twice a week *ad libitum*. We had 8 worker groups for each of the 3 treatments for each of the 3 species. After 3 weeks, 1 forager per worker group was taken per acclimation treatment and species (8 groups × 3 treatments × 3 species). Due to high worker mortality, we could only sample a forager from 4 instead of 8 worker groups in the 20 °C treatment of *L. brunneus*. All individuals were dissected on ice, and the last 2 segments of the gaster with the fat body and epidermal cells attached were put in TRIzol (Thermo Fisher) and stored at −80 °C. RNA was extracted using the Direct-zol Miniprep kit (Zymo), including a DNAse-treatment. RNA quality assessment, quantification, library preparation, and 150 bp paired-end sequencing on a Novaseq 6000 were conducted at NovoGene.

### Gene expression and gene network analyses

Raw read quality was checked using FastQC version 0.11.8 (Andrews 2010), and reads were trimmed using TRIMMOMATIC 2.8.4 (Bolger et al. 2014) with the following parameter settings: ILLUMINACLIP: adapter_3.0.fa:2:5:5:6:true LEADING:7 SLIDINGWINDOW:4:20 MINLEN:100 TOPHRED33 (Supplementary 1: Table 1). Reads of all 3 species were mapped to the *L. niger* reference genome (GCA_001045655.1) using HiSat2 v2.1.0 (Kim et al. 2015). Since mapping rates for *L. brunneus* were low, we changed the SNP score to –mp 4,2 for this species only. Read counts were obtained using HTSeq v0.13.5 (Anders et al. 2015) (Supplementary 1: Table 1). We opted for the single reference genome approach since only the *L. niger* genome was available at the time of analysis, and the reference genome approach seems superior to *de novo* transcriptomes, even if the genome comes from a closely related species (Ockendon et al. 2016; Marchant et al. 2026). Overall gene expression was compared among species and acclimation treatments using a PERMANOVA (command *adonis2*, package *vegan*), with 9,999 permutations, and colony as a factor nested in species. Here, we used the normalized complete read counts table (generated using the *DESeq2* function, R package *DESeq2*, Love et al. 2014) as input. As *L. brunneus* differed strongly from the others, we then ran separate PERMANOVAs for each acclimation treatment, including *L. niger* and *L. platythorax* only. We used the *Wald test* in the R package *DESeq2* (Love et al. 2014) to conduct pairwise comparisons within species between the 3 treatments to identify differentially expressed genes (DEGs). Significance was assessed at a false discovery rate (FDR) < 0.05, using the Benjamini–Hochberg method for multiple testing correction as implemented in *DESeq2*. We then determined the overlap between the species-specific DEGs to make inferences on the differences in acclimation response to the different treatments by constructing Venn diagrams (https://bioinfogp.cnb.csic.es/tools/venny/). For each species, the numbers of upregulated genes were compared between the pairs of acclimation treatments using χ^2^ tests. Furthermore, we used χ^2^ tests to analyze, for the 20 °C to 28 °C contrast, whether each pair of species shared more or less DEGs than expected by chance. For functional annotation, we performed GO enrichment analyses with *TopGO* v. 2.28.0 (Alexa and Rahnenfuhrer 2023) using Fisher's exact test to test for enrichment of GO terms. We furthermore conducted weighted gene coexpression network analyses *WGCNA* v1.64-1 (Langfelder and Horvath 2008), with a soft threshold of 4, 5, and 5 for *L. brunneus, L. niger,* and *L. plathythorax,* respectively, to determine sets of coexpressed gene modules, followed by an *hdWGCNA* (Morabito et al. 2023) to test for module preservation between species pairs (using *t* tests to compare *z* scores). Using Fisher's exact tests, we analyzed which modules contained more or less CHC-candidate genes than expected by chance. All analyses were done in R version 4.0.4 (R Core Team 2020). Scripts for the bioinformatic analyses are available in the online supplement of this manuscript compiled in “Supplementary 3,” available at DOI 10.5281/zenodo.18847845. Tables with all results can be found in Supplementary 1.

### Correlation of expression patterns

As a second means of informing about (dis-)similarity of expression patterns between species, we investigated the correlations of directions and effect sizes (log fold changes) of expression across species. We extracted log2FC values for each of the 3 contrasts and conducted Pearson's correlations in R version 4.0.4 (R Core Team 2020). Since the large number of data points will inevitably lead to a significant result, we ran an additional Bayesian correlation analysis. We employed a Bayesian multivariate regression model using the brms package (version 2.22.0; Bürkner 2018). The model was fit with a joint response of the log2​ fold change (log2FC) values for both species. We used a standard Gaussian likelihood and a default prior for the residual correlation parameter. We ran 4 Markov Chain Monte Carlo (MCMC) chains for 4,000 iterations, with 2,000 warm-up iterations, to ensure convergence. The Bayesian results corroborate Pearson's correlation and are summarized in the supplement (Supplementary 1: Table 2).

### Identification of CHC candidates

We conducted a thorough literature search on Web of Science in December 2024 to identify publications reporting on identified and functionally tested cuticular hydrocarbon synthesis genes (Supplementary 1: Table 3) using the search terms “(CHC OR cuticular hydrocarbon)” AND synthesis AND gene. We extracted gene IDs from these publications and downloaded the corresponding nucleotide or protein sequences from the corresponding repository. Nucleotide sequences were translated into amino acid sequences using TransDecoder v5.1.0 (http://transdecoder.sf.net), and only the longest open reading frame was retained. We used this list of 98 candidate sequences from 6 insect species to conduct, for each, a reciprocal local BLAST search with the predicted protein sequences encoded by the *L. niger* genome. Since many of the CHC candidates obtained from the various publications were not annotated, and/or their specific function (except for being involved in CHC synthesis) was unknown, we decided to create a phylogeny of all candidate sequences plus the *Lasius* sequences we identified via the blast search vs the CHC candidates (Supplementary 4). To infer orthology of candidates, Clustal Omega on the web (Madeira et al. 2022) was used to construct a multiple sequence alignment, and the IQ-Tree web server (Trifinopoulos et al. 2016) was used to construct a maximum likelihood tree using standard settings. The consensus tree was visualized using FigTree v1.4.4 (Rambaut 2010). Please note that this phylogeny is not intended to provide reliable information about the phylogenetic relationships across sequences, but it allows clustering sequences according to their similarity and membership to broader gene families such as synthases or elongases.

### Text editing

ChatGPT-v5.2 (OpenAI) was used to shorten the introduction and discussion and to correct grammar and improve syntax throughout the text. Suggestions from ChatGPT were reviewed and validated to ensure accuracy and integrity of the content.

## Results

### Overview of RNA-seq output

We obtained between 238 to 403 Mio. total raw reads per sample, of which 220 to 371 Mio. paired-reads remained after trimming (Supplementary 1: Table 4). Mean alignment rates ranged from 84% for *L. brunneus* to 91% in *L. platythorax*, to 93% in *L. niger*. To check whether differences in alignment rate could affect the numbers of detected DEGs, we ran an additional *DESeq2* analysis for *L. niger* with 10% fewer reads. This yielded 5 and 2 differentially expressed genes more in the 20 to 28 and 20-F contrast, respectively, and 2 genes less in the 28-F contrast, suggesting that our results are robust to the differences in alignment rate (Supplementary 1: Table 5). As a further check, we extracted the read counts of DEGs of all 3 species to test whether *L. brunneus* might not have had sufficient mapped reads at those genes to infer significance, such that DEGs might have been missed (Supplementary 2: Fig. 3). A Kruskal-Wallis test across all species indeed indicated differences in read counts of the DEGs (chi^2^ = 7.25, df = 2, *P* = 0.027). However, pairwise post-hoc Mann-Whitney U tests only indicated differences between *L. brunneus* and *L. niger* (*P* = 0.014), but not between *L. brunneus* and *L. platythorax* (*P* = 0.728) or *L. niger* and *L. platythorax* (*P* = 0.122). There is thus no systematically lower read count of *L. brunneus* compared to the other 2 species, and hence, there was no evidence that the different backmapping rates biased our results.

### Global gene expression: effects of species and climate regime

**Fig. 1.**
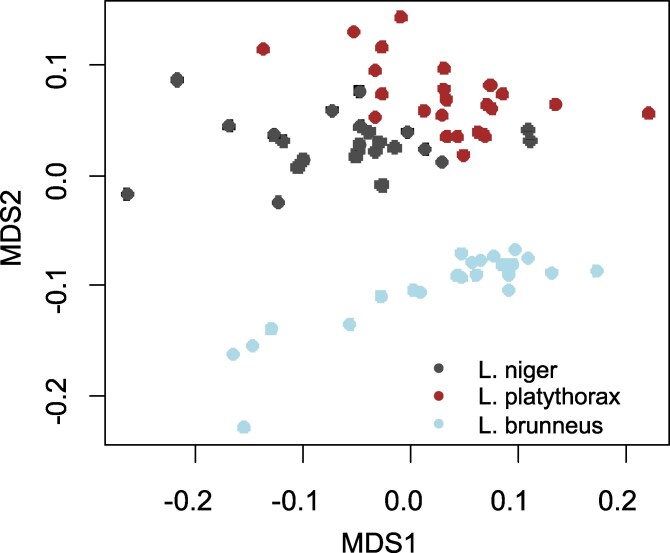
Overall gene expression patterns in the 3 *Lasius* species. The graph shows an NMDS ordination based on the raw read counts of the species. Since the PERMANOVA indicated only a species, but no temperature effect, only species are color-coded differently here.

Global gene expression varied most strongly between species (PERMANOVA: F_2_ = 10.6, r^2^ = 0.20, *P* = 0.0001), followed by treatment (F_2_ = 1.8, r^2^ = 0.034, *P* = 0.029) and a marginally nonsignificant species:treatment interaction (F_4_ = 1.4, r^2^ = 0.054, *P* = 0.069) (Fig. 1). *L. brunneus* differed most strongly from the other 2 species (Fig. 1). Therefore, we ran an additional analysis only including the sister species *L. niger* and *L. platythorax* and compared them separately for each acclimation treatment. Here, *L. niger* and *L. platythorax* differed most strongly in the fluctuating treatment (F_1_ = 2.9, r^2^ = 0.17, *P* = 0.0001) and (albeit less) in the 20 °C treatment (F_1_ = 2.8, r^2^ = 0.17, *P* = 0.0075), while in the 28 °C treatment, overall gene expression did not differ (F_1_ = 1.5, r^2^ = 0.095, *P* = 0.16). Thus, gene expression was species-specific for *L. niger* and *platythorax*, but not if they were acclimated to warm conditions.

**Fig. 2.**
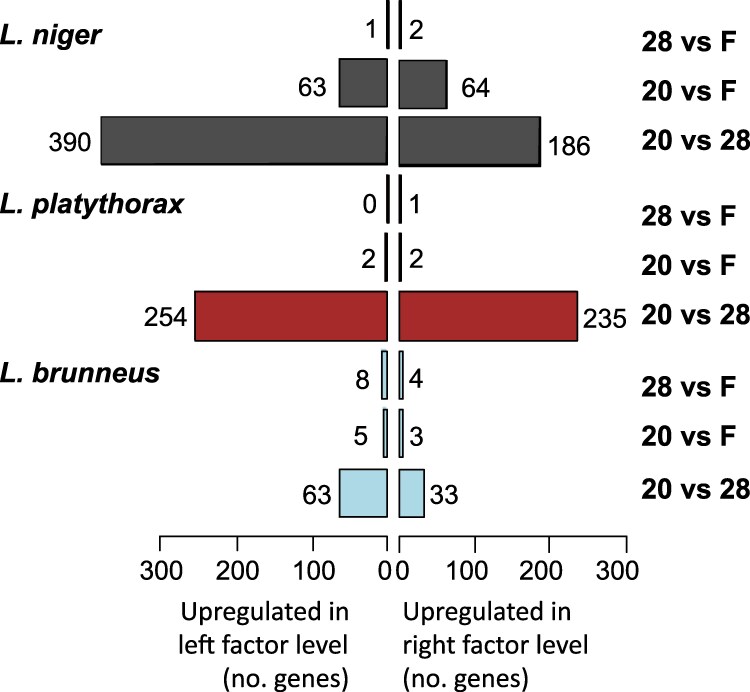
Numbers of differentially expressed genes (DEGs) per species and treatment. The bars indicate the numbers of genes with higher expression in pairwise comparisons of acclimation treatments within each species. Per species, 3 pairwise contrasts are given to the right of the plot (“28 vs F”: constant 28 °C vs fluctuating; “20 vs F”: constant 20 °C vs fluctuating; “20 vs 28”: constant 20 °C vs constant 28 °C). For example, for “20 vs 28” the left factor level is 20 °C, and the right factor level is 28 °C.

Following results from Baumgart et al. (2022), where the acclimation response was largest in *L. niger* and lowest in *L. brunneus*, in terms of changes in CHC profile and drought survival, we expected the highest transcriptional plasticity in *L. niger*, which occupies open habitats with large daily temperature variation, and the lowest in *L. brunneus* (Sandoval-Castillo et al. 2020). Indeed, pairwise contrasts between the 3 treatments within species revealed between 116 in *L. brunneus* and 706 differentially expressed genes (DEGs) in *L. niger*  (Fig. 2; Table 1; Supplementary 1: Table 6). We tentatively conclude that *L. brunneus* is the least plastic among the 3 and concomitantly has the lowest capability to change gene expression in response to changing temperature conditions.

**Table 1.** Summary statistics of differentially expressed genes between acclimation treatments per species. DEGs = Number of differentially expressed genes per contrast. “hi” = The number of higher expressed genes in the respective acclimation treatment. fl = Fluctuating temperature regime.

Species	Contrast	DEGs	hi-20	hi-28	hi-fl	Enriched GO
*L. brunneus*	20_28	96	63	33		28
	20_F	8	5		3	7
	28_F	12		8	4	3
*L. niger*	20_28	576	380	186		69
	20_F	127	63		64	16
	28_F	3		1	2	NA
*L. platythorax*	20_28	489	254	235		50
	20_F	4	2		2	NA
	28_F	1		0	1	NA

We furthermore expected a stronger transcriptional response in the fluctuating temperature regime, consistent with increased regulatory demands imposed by thermal variability (Podrabsky and Somero 2004; Colinet et al. 2015). However, the highest number of DEGs (1,176 (88%) of 1,316 DEGs in total) was found between the 20 °C and 28 °C acclimation treatments in all 3 species (576, 489, and 99 in *L. niger, L. platythorax, and L. brunneus*, respectively; Fig. 2; Table 1; Supplementary 1: Table 6), indicating the largest difference between these 2 constant temperature conditions. Considerably less genes were differently expressed between the fluctuating treatment and both of the constant treatments. In the 28 °C-fluctuating contrast, there was only 1 DEG in *L. platythorax*, *contactin-associated 2,* 3 DEG in *L. niger*, and 12 in *L. brunneus*. In the 20 °C-fluctuating contrast, only 4 genes were differentially expressed in *L. platythorax* and 8 in *L. brunneus,* but 127 in *L. niger*. Interestingly, more genes showed higher expression in the 20 °C treatment than in the 28 °C treatment (Fig. 2). This was true for *L. niger* (χ^2^ test: $\chi_{1}^{2}$ = 66.5, *P* < 0.0001) and *L. brunneus* ($\chi_{1}^{2}$ = 9.4, *P* = 0.0022), but not for *L. platythorax* ($\chi_{1}^{2}$ = 0.7, *P* = 0.39).

Genes differentially expressed in the 20 °C to 28 °C contrast of *L. niger* and *L. platythorax* were characterized by transcription factor, histone, chromatin, and transposon-associated genes involved in gene regulation, plus methyltransferases in *L. platythorax* (Table 1). In *L. brunneus*, we found transcription factor, histone, and chromatin-associated genes, but at much lower numbers than in the other 2 species. In all 3 species, we identified several fatty acid synthesis-associated genes, such as dehydrogenases and esterases in *L. niger*, synthase, reductase, and hydrolase in *L. platythorax*, as well as elongation factors and desaturases in *L. brunneus*. In *L. platythorax*, we found several cuticle-associated genes, the same for *L. niger,* with additional chitin genes. In the latter 2 species, we also found *odorant receptor isoform a* differentially expressed, as well as *gustatory receptor 28b-like protein* and *olfactomedin-like protein 2a protein* in *L. niger*.

Based on our experimental setup, there are 3 technical sources that could have influenced the results. We address these below and explain why we believe they play no, or only a marginal role, and why we believe our results are still reliable. First, we collected the individuals after 3 weeks of acclimation to the appropriate regime. It is thus possible that major acclimatory and thus gene regulatory changes happened in the first few days of acclimation, such that only small (thus hardly detectable) gene regulatory changes are necessary to maintain them. However, the aim of this study was to investigate long-term acclimation effects, not short-term acute responses. Second, we used a single reference genome, and *L. brunneus* shows lower backmapping rates than the other species. However, our downsampling approach showed only a marginal effect (±5 DEGs), and comparable read-counts between species at differentially expressed genes. Moreover, a previous accompanying study showed that acclimation did not alter the *L. brunneus* drought survival, corroborating the low expression response found here (Baumgart et al. 2022). Third, we collected all samples in the morning (closer to 20 °C), which could have led to a bias in the results, including the fluctuating regime. However, we found the contrasts 20 °C-fluctuating and 28 °C-fluctuating to have comparable numbers of DEGs in *L. platythorax* and *L. brunneus* (respectively). In *L. niger,* there were even more DEGs in the 20 °C-fluctuating contrast than in the 28 °C-fluctuating one. This indicates that the precise time point of sampling was less relevant in our study and confirms that differences in gene expression are due to the acclimation regimes rather than the temperature at the time of sampling.

### Functional enrichment analysis highlights the role of lipid metabolism associated with temperature acclimation

**Fig. 3.**
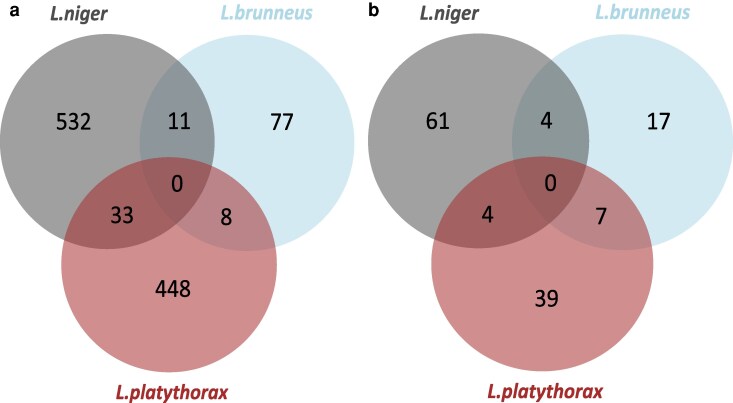
Overlap of (a) differentially expressed genes and (b) enriched functions among species. The Venn diagrams depict the differentially expressed genes in the 20 °C/28 °C contrast. Panel (a) shows the number of private and overlapping genes among species for this contrast. Panel (b) shows the overlap in significantly enriched GO terms of those genes.

Functional enrichment analyses of differentially expressed genes within species revealed between 0 and 69 overrepresented functions per contrast (Table 1; Supplementary 1: Table 7). In *L. platythorax,* functions associated to (i) biosynthesis such as “biosynthetic process,” “organic substance biosynthetic process,” (ii) lipids “(phosphor-)lipid metabolic process,” but also (iii) gene regulatory functions such as “methylation,” “chromatin organization,” and “gene expression” were overrepresented among DEGs in the 20 to 28 °C contrast. In *L. niger*, we found, for example, “oxidation reduction” and “monosaccharide metabolic process,” and in *L. brunneus,* “lipid phosphorylation.” *L. niger* shared 4 enriched functions with each of the other species, which, in turn, shared 7 (Fig. 3b). The shared functions of *L. platythorax* and *L. niger* include “alcohol biosynthetic process” and “RNA metabolic process,” and “lipid phosphorlyation” and “cellular metabolic processes” in *L. platythorax* and *L. brunneus* (Supplementary 1: Table 5). Acclimation to different temperature conditions led to changes in fatty acid composition (Podrabsky and Somero 2004; van Dooremalen et al. 2011), as reflected in our results, where lipid-associated functions were repeatedly enriched across multiple comparisons.

### Little overlap of DEGs among species

To test whether acclimatory responses differed between species, we analyzed “within-species” DEGs between acclimation treatments and compared them among species. We expected relatively similar expression responses across species. However, the comparison revealed that there was only overlap between at most 2 species in the 20 to 28 °C contrast (Fig. 3a), and most DEGs were restricted to a single species (Supplementary 1: Table 8). Concerning the 20 °C to 28 °C contrast, 8 differentially expressed genes were shared by *L. brunneus* and *L. platythorax*, 11 by *L. brunneus* and *L. niger,* and 33 by *L. niger* and *L. platythorax* (Supplementary 1: Table 8). This is less than expected by chance (*L.nig.-L.pla.*: $\chi_{1}^{2}$ = 142.74, *P* < 2.2e−16; *L.nig-L.bru.*: $\chi_{1}^{2}$ = 37.789, *P* = 7.9e−10; *L.pla.-L.bru.*: $\chi_{1}^{2}$ = 41.29, *P* = 1.3e−10; Fig. 3a). The 33 genes shared between *L. niger* and *L. platythorax* included genes associated with perception, gene regulation, and stress response, such as the *odorant receptor isoform a*, *cuticular protein analogous to peritrophins 3-b precursor*, *histone h4 transcription factor-like protein*, *methyltransferase family member.1*, *activator of 90 kda heat shock protein atpase 1-like protein*, and *cold shock domain-containing protein 1*. Among the genes that were differentially expressed between the fluctuating treatment and either 20 °C or 28 °C, none overlapped between any pair of species, that is, all were species-specific (Supplementary 1: Table 8).

### CHC biosynthesis gene expression is mainly species-specific

CHC synthesis is derived from the fatty acid metabolism, and CHC synthesis genes are part of fatty acid synthesis gene families (Dembeck et al. 2015; Holze et al. 2021; Sprenger et al. 2021). Elongases and desaturases are extremely variable and have expanded dramatically in social insects, especially in ants (Hartke et al. 2019). This is corroborated by the results of this study. A total of 116 *L. niger* genome-derived protein sequences resulted in a positive BLAST hit against the 98 CHC biosynthesis genes obtained from our literature search. To determine putative orthology of candidates, we constructed a maximum likelihood tree including these 98 genes plus the 116 *L. niger* hits (Supplementary 2: Fig. 1). Out of 1,180 unique DEGs across species, 7 matched 1 of the 116 *L. niger* CHC synthesis candidates (Table 2). They included 3 reductases, 1 desaturase, 1 CytP450, 1 hydrolase, and 1 NADH dehydrogenase. Moreover, we found evidence of a large fatty acid synthase expansion in *L. niger* (Supplementary 2: Fig. 1).

**Table 2.** List of the 7 differentially expressed candidate genes.

*L. niger* proteinID	Gene family	Contrast	Higher in	Species
KMQ91077.1	NADH	20_28	28	*L. brunneus*
KMQ91667.1	Desaturase	20_28; 20_fl	fluctuating	*L. brunneus*
KMQ86903.1	Reductase	20_28	20	*L. platythorax*
KMQ92013.1	Reductase	20_28; 20_fl	20; 20	*L. niger*
KMQ92849.1	Reductase	20_28	28; fl	*L. niger*
KMQ98367.1	CytP450	20_28	20	*L. niger*
KMQ87163.1	Hydrolase	20_fl	20	*L. niger*

*L. brunneus* was the only of the 3 species that produces major percentages of unsaturated compounds, whose biosynthesis requires desaturases (Baumgart et al. 2022). Indeed, the only desaturase (KMQ91667.1) that was differentially expressed was found in this species. Here, it was higher expressed at 20 °C, both compared to 28 °C and to the fluctuating treatment. Desaturases in Moris et al. (2023) neither produced clear CHC effects (knock-down of GB51236) nor resulted in a decrease of alkenes with even double-bond position (knock-down of GB51238; i.e. high expression of GB51238 results is high alkene abundance). In our study, KMQ91667.1 was highest expressed in the 20 °C treatment, concomitant with lowest alkene and alkadiene abundances, but highest abundances of methyl-branched alkenes. The majority of *n*-alkenes in *L. brunneus* had double bonds at even positions as found by DMDS derivatization (FM unpublished data). However, since our candidate is not a 1:1 ortholog, the precise function of this gene is speculative. We expected to find CHC-candidate genes associated with *n*-alkanes and methyl-branched alkanes to be similarly differentially expressed between species, as these 2 CHC classes commonly shift in abundance under different temperature regimes (Sprenger and Menzel 2020; Baumgart et al. 2022). However, we observed species-specific expression patterns of CHC-candidate genes, which could be caused by the distinct baseline CHC profiles between species and the mainly species-specific changes observed following acclimation to different temperature treatments (Baumgart et al. 2022).

### Global expression patterns are slightly correlated

**Fig. 4.**
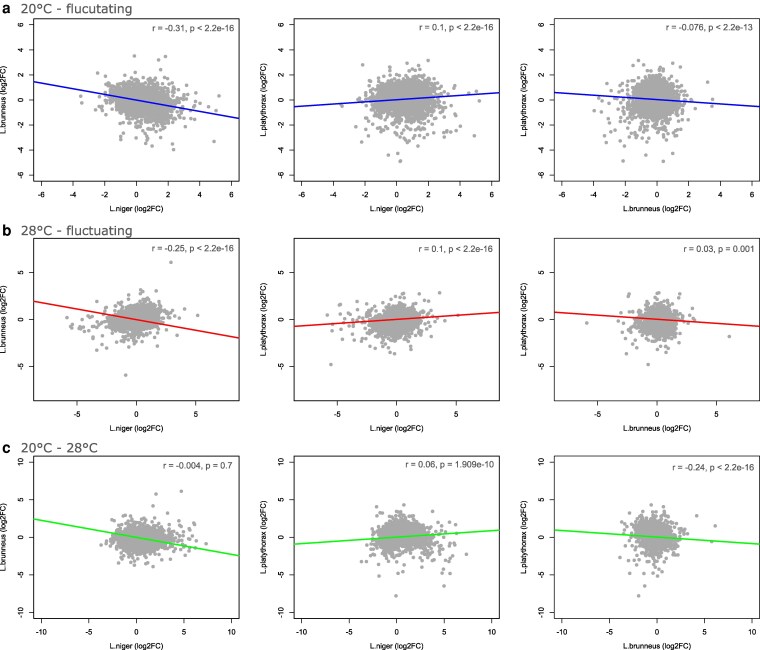
Correlation of log2FC values between pairs of species according to pairwise contrasts of the different acclimation treatments. Rows represent the different contrasts, while each column depicts one of the according species pair. Pearson's correlation statistics are given in the upper right corner of each plot.

In addition to categorical differential expression analyses, we compared the magnitude and direction of log2FC across species to investigate genome-wide trends in expression direction and magnitude and to detect shared response patterns. Global gene expression patterns (log2FC) between species were subtly, but significantly correlated. The correlation coefficients were low, ranging from −0.3 to +0.24, often being close to zero (Fig. 4; Supplementary 1: Table 2), which may be due to generally low log2FC values, with the majority ranging between +/− 5 (Fig. 4). Only *L. brunneus* and *L. niger* log2FC values from the 20 °C to 28 °C contrast do not correlate. The orientation of the slopes (negative or positive) is always the same within each species pair across contrasts. While *L. niger* and *L. platythorax* share a positive relationship, it is negative in the other 2 pairs containing *L. brunneus*. This may indicate that *L. niger* and *L. platythorax* share a conserved transcriptional response architecture, that is, show similar coping strategies. The correlations of those 2 species with *L. brunneus* are negative and may thus indicate that *L. brunneus* indeed follows a different response strategy to temperature acclimation. This may also suggest *L. brunneus* constraints in response to different temperature regimes following the missing phenotypic response observed in Baumgart et al. (2022).

### Gene networks are preserved across species, especially the ones containing many CHC-candidate genes

Finally, we conducted a gene network analysis to investigate gene coexpression patterns with respect to temperature treatments and CHC genes. In *L. niger* and *L. platythorax*, 6 and 3 modules (respectively) were significantly associated with temperature treatment (Supplementary 1: Table 9). In *L. brunneus*, there were no temperature-associated modules.

Five of the 6 temperature-associated modules in *L. niger* were enriched for oxidation reduction processes. The *tan* module, which contained 3 of the CHC candidates, was enriched for lipid metabolic processes, reactive oxygen species, and small metabolic processes, among others (Supplementary 1: Table 7). In *L. platythorax*, one of the 3 temperature-associated modules (*red*) was enriched for several lipid-associated functions, such as lipid biosynthesis process, lipid metabolic process, and lipoprotein biosynthetic process. The module *Salmon* was enriched for multiple regulation-associated functions (Supplementary 1: Table 9).

An over- or under-representation of the 116 CHC-candidate genes was found in 1 to 3 modules per species. The module with the most CHC genes contained 40.2%, 29.4%, and 22.4% of all CHC genes found in the respective species in *L. platythorax* (Fisher test: *P* < 0.0001), *L. niger* (*P* < 0.0001), and *L. brunneus* (*P* = 0.00011), respectively. In all 3 species, modules with overrepresented CHC genes were enriched in lipid-associated functions and moleculer metabolic processes. Modules with under-representation of CHC candidates were enriched for regulatory and signal transduction functions. In *L. platythorax*, the module with 40% of all CHC candidates had enriched functions that included lipid biosynthetic process, lipid metabolic processes, and response to heat (Supplementary 1: Table 10). The expression pattern of genes within this module was conserved across all 3 species (Supplementary 1: Table 11).

Temperature-associated modules often contained CHC-candidate genes. In *L. niger*, 10 CHC-candidate genes were found in temperature-associated modules (0–5 per module); 3 of them were also differentially expressed (Table 3). However, the number of CHC genes in each temperature-associated module never differed from random expectation (Fisher tests: all *P* > 0.24, Table S10). In *L. platythorax,* however, 1 temperature-associated module contained 5 CHC candidates, 2 of which were differentially expressed. This is a much lower number of CHC genes than expected from random (Fisher test: *P* = 0.00018). One other module contained another single candidate gene, and the third temperature-associated one contained no CHC genes (deviation from random: both *P* > 0.63). In both ant species, all CHC candidates in the temperature-associated modules were species-specific (Supplementary 1: Table 9).

**Table 3.** CHC candidates in gene coexpression modules significantly associated with temperature. Differentially expressed CHC-candidate genes are shown in bold. No modules were associated with temperature in *L. brunneus*. “Direct neighbor” indicates whether the according *L. niger* protein is a direct ortholog to one of the functionally annotated CHC-candidate genes obtained from literature (Supplementary 1: Table 3; Supplementary 2: Supplementary Fig. 1).

Significant modules	CHC candidates	Direct neighbor
*L. niger*		
Green	KMQ82598.1	
Green	KMQ90794.1	Yes
Green	**KMQ92849.1**	
Green	KMQ93814.1	Yes
Green	KMQ96383.1	Yes
Light green	**KMQ92013.1**	
Light green	KMQ93645.1	yes
Tan	KMQ86151.1	yes
Tan	**KMQ87163.1**	
Tan	KMQ91967.1	
*L. platythorax*		
Red	KMQ95418.1	yes
Turquoise	KMQ85969.1	
Turquoise	**KMQ86903.1**	
Turquoise	KMQ96835.1	
Turquoise	KMQ97037.1	
Turquoise	**KMQ98367.1**	

**Fig. 5.**
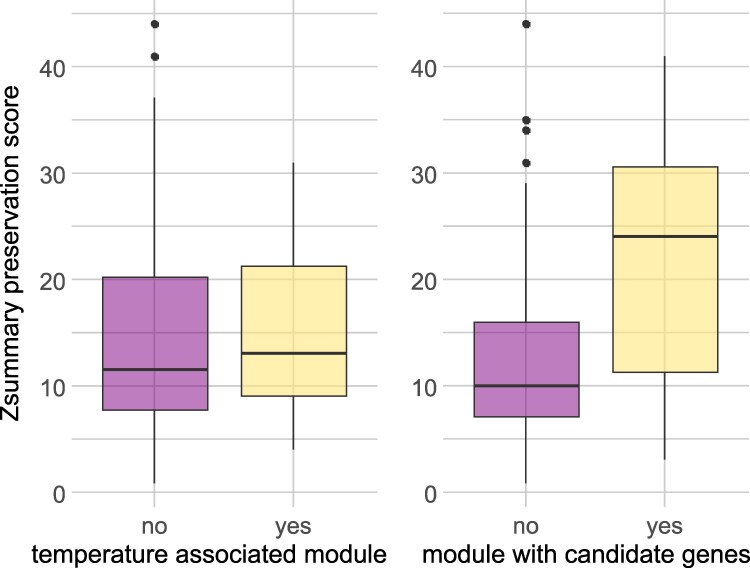
Gene network preservation. The boxplots show *zsps* scores, which indicate the degree of similarity of gene coexpression across species. The *zsps* scores are depicted for modules significantly associated or not associated with temperature regime, as well as for modules that do or do not contain CHC-candidate genes.

In general, 8 to 14 modules were preserved (Z-summary preservation score (*zsps*) > 10) across species in reciprocal pairwise comparisons and reached values up to zsps = 44 (Supplementary 1: Table 11). The mean *zsps* as a measure of the strength of the similarity in gene content and expression between modules between species pairs did not differ between the 3 species (F_2_ = 2.83, *P* = 0.06), taking only the values from the reference species into account. Temperature-associated modules were not more preserved (higher *zsps*) than nonassociated ones (t = −0.20, df = 27.8, *P* = 0.85; Fig. 5a). However, modules with overrepresentation of differentially expressed CHC genes were more preserved than those without CHC candidates (t = −3.97, df = 34.89, *P* = 0.00035; Fig. 5b). We also tested whether the 2 sister species were more similar in terms of module preservation than they are to *L. brunneus*, but there were no *zsps* differences between species pairs (F_2_ = 0.49, *P* = 0.62; Supplementary 2: Fig. 2). In summary, gene coexpression networks associated with temperature acclimation were conserved across species, putatively reflecting shared regulatory architectures shaped by common ancestry. We identified several modules with an overrepresentation of CHC-candidate genes, which might reflect the necessity to coregulate many of those genes in order to express and adjust species-specific CHC profiles. Modules associated with temperature acclimation, however, contained only a few CHC candidates, indicating that they only play a minor role in the overall acclimation response.

## Discussion

Organisms must cope with temperature variation on a daily and annual scale. Acclimation is a key component of phenotypic plasticity, enabling persistence under variable conditions. Here, we investigated whether acclimatory responses differ among 3 ant species in number, identity, function, and coexpression of differentially expressed genes. Differences in acclimatory plasticity may reveal their ability to cope with climate change-induced stressors.

Despite the strong temperature dependence of ectotherm metabolism, acclimation resulted in relatively subtle transcriptomic responses. Across species, only a few hundred genes were differentially expressed, with the strongest contrasts consistently observed between constant 20 °C and constant 28 °C. This was unexpected, given pronounced acclimatory effects previously reported in all 3 species, including changes in CHC profiles and (except for *L. brunneus*) enhanced drought survival following warm or fluctuating acclimation. Thus, substantial phenotypic plasticity appears to be mediated by limited but targeted changes in gene expression.

We expected the fluctuating temperature regime to induce stronger transcriptional responses than constant regimes, consistent with the increased regulatory demands imposed by thermal variability (Podrabsky and Somero 2004). However, the fluctuating temperature regime induced remarkably few transcriptional changes relative to either constant treatment. We interpret this as an intermediate response combining properties of both constant regimes, resulting in limited divergence from either condition at the transcriptomic level. This contrasts with studies in other taxa, such as killifish, where fluctuating temperatures elicited the strongest gene expression responses (Podrabsky and Somero 2004). In *Lasius* ants, however, fluctuating acclimation also conferred drought resistance comparable to warm acclimation, supporting the notion that fluctuating environments may promote a generalist phenotype without requiring extensive transcriptional remodeling.

As expected, *L. niger*, which inhabits open and thus climatically variable habitats (Renaud et al. 2011), showed the highest acclimatory responses and thus the highest plasticity. In contrast, *L. brunneus* exhibited the weakest gene expression response to acclimation. While technical factors cannot be fully excluded, this finding is supported by phenotypic data showing no acclimation-induced improvement in drought resistance. Together, these results suggest reduced plasticity in *L. brunneus*, potentially increasing its vulnerability to rising temperatures and drought. Low plasticity has been identified as a risk factor for climate change sensitivity (Diamond et al. 2012), highlighting its relevance for predicting species-specific responses.

Analyses of CHC-associated candidate genes revealed extensive gene family expansion, particularly for fatty acid synthases in *L. niger*. Most CHC candidates were not associated with an acclimation-responsive coexpression module, and only a few were differentially expressed. This suggests that species-specific CHC profiles are largely maintained by constitutive expression, with modulation of only a small subset of genes sufficient to induce acclimation-related CHC changes. Highly conserved coexpression networks across species further indicate that global transcriptional programs are largely shared, while species-specific phenotypic responses arise from small expression differences.

Overall, our results demonstrate that acclimatory responses in *Lasius* ants involve subtle, species-specific transcriptional changes embedded within highly conserved gene networks. Differences in plasticity, rather than overall metabolic architecture, appear to underlie differential resilience to environmental change. Integrating plasticity with genomic diversity will be critical for predicting species vulnerability to climate change and informing conservation strategies.

## Data Availability

All sequence data were uploaded to ENA and can be accessed via the project accession number PRJEB76825. Supplementary material and scripts are available via DOI 10.5281/zenodo.18847845.
